# Myocardial Viability Imaging using Manganese‐Enhanced MRI in the First Hours after Myocardial Infarction

**DOI:** 10.1002/advs.202003987

**Published:** 2021-04-02

**Authors:** Nur Hayati Jasmin, May Zaw Thin, Robert D. Johnson, Laurence H. Jackson, Thomas A. Roberts, Anna L. David, Mark F. Lythgoe, Philip C. Yang, Sean M. Davidson, Patrizia Camelliti, Daniel J. Stuckey

**Affiliations:** ^1^ UCL Centre for Advanced Biomedical Imaging Division of Medicine University College London London WC1E 6DD UK; ^2^ School of Medical Imaging Faculty of Health Sciences Universiti Sultan Zainal Abidin Kuala Terengganu 21300 Malaysia; ^3^ School of Biosciences and Medicine University of Surrey Guildford GU2 7XH UK; ^4^ School of Biomedical Engineering & Imaging Sciences King's College London London SE1 7EH UK; ^5^ UCL Elizabeth Garrett Anderson Institute for Women's Health London WC1E 6BT UK; ^6^ Division of Cardiovascular Medicine Department of Medicine Stanford University Stanford CA 94305 USA; ^7^ The Hatter Cardiovascular Institute University College London 67 Chenies Mews London WC1E 6HX UK

**Keywords:** imaging, manganese, MRI, myocardial infarction, viability

## Abstract

Early measurements of tissue viability after myocardial infarction (MI) are essential for accurate diagnosis and treatment planning but are challenging to obtain. Here, manganese, a calcium analogue and clinically approved magnetic resonance imaging (MRI) contrast agent, is used as an imaging biomarker of myocardial viability in the first hours after experimental MI. Safe Mn^2+^ dosing is confirmed by measuring in vitro beating rates, calcium transients, and action potentials in cardiomyocytes, and in vivo heart rates and cardiac contractility in mice. Quantitative T1 mapping‐manganese‐enhanced MRI (MEMRI) reveals elevated and increasing Mn^2+^ uptake in viable myocardium remote from the infarct, suggesting MEMRI offers a quantitative biomarker of cardiac inotropy. MEMRI evaluation of infarct size at 1 h, 1 and 14 days after MI quantifies myocardial viability earlier than the current gold‐standard technique, late‐gadolinium‐enhanced MRI. These data, coupled with the re‐emergence of clinical Mn^2+^‐based contrast agents open the possibility of using MEMRI for direct evaluation of myocardial viability early after ischemic onset in patients.

## Graphical abstract

Early measurements of tissue viability after myocardial infarction (MI) are essential for accurate diagnosis and treatment planning. Here, the safety and efficacy of manganese‐enhanced magnetic resonance imaging (MEMRI) in vitro in cardiomyocytes and in vivo in mouse models of MI are evaluated. It is demonstrated that MEMRI offers a novel biomarker of myocardial viability in the first hours after MI.

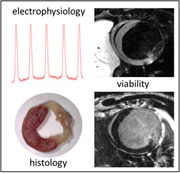

## 1 Introduction

Cardiac imaging has revolutionized our ability to diagnose heart disease, quantify mechanisms of pathology, and determine optimal therapy in patients.^[^ ^1^ ^]^ Current techniques can interrogate structure, contractility, metabolism, and fibrosis^[^ ^2^, ^3^, ^4^ ^]^ but struggle to directly quantify one of the most important determinants of patient morbidity, myocardial viability.^[^ ^5^, ^6^, ^7^, ^8^, ^9^ ^]^

Late gadolinium‐enhanced magnetic resonance imaging (LGE‐MRI) is routinely used clinically to assess myocardial damage.^[^ ^10^ ^]^ Despite LGE‐MRI being one of the most important diagnostic and prognostic measures of infarct size,^[^ ^1^, ^2^, ^11^ ^]^ it has several limitations: 1) chelated Gd^3+^ accumulates nonspecifically in the infarct owing to a mixture of cell membrane rupture, increased extracellular space, edema, and perfusion deficits, meaning changes in image contrast relate to several different tissue properties,^[^ ^12^ ^]^ 2) infarct‐related increases of extracellular space and edema develop at variable rates and over several hours after damage, compromising the accuracy of LGE‐MRI early in acute myocardial infarction (AMI),^[^ ^13^ ^]^ 3) concerns over the safety of Gd‐based contrast agents have been raised owing to its links to nephrogenic systemic fibrosis^[^ ^14^ ^]^ and accumulation in the brain.^[^ ^15^ ^]^ MRI can also be used to evaluate tissue damage through increased signal in T2‐weighted images derived from increased water content in jeopardized myocardium. However, myocardial edema progressively develops in a bimodal pattern after injury, meaning accurate assessment of viability is challenging.^[^ ^16^ ^]^

Alternatively, positron emission tomography (PET) can evaluate myocardial metabolism through the accumulation of the radioactive glucose analogue ^18^F fluorodeoxyglucose (FDG) within metabolically active cells. This approach provides a more direct evaluation of viability than LGE‐MRI, but specificity can be hindered by high FDG uptake in the immune cells which are prevalent within the infarcted tissue. In addition, fasting and fatty acid lowering protocols are required to induce a shift to glucose metabolism and ensure reproducible and sufficient FDG uptake,^[^ ^17^ ^]^ meaning ^18^F‐FDG‐PET cannot be performed in the initial hours after a patient presents with suspected MI. Further, trials have shown that clinical decisions assisted by ^18^F‐FDG‐PET do not alter patient outcome.^[^ ^8^, ^18^ ^]^ Single‐photon emission computed tomography (SPECT) perfusion scans with ^99m^Tc Sestamibi and ^201^TI can be acquired in AMI, but are low resolution, often require a second redistribution imaging session,^[^ ^19^ ^]^ and have not enabled the identification of patients with greater likelihood of survival.^[^ ^7^ ^]^

One property that is highly sensitive to altered myocardial viability and changes rapidly after damage is calcium handling. Calcium is integral to cardiomyocyte contraction and alterations to calcium uptake and handling are present in many cardiomyopathies.^[^ ^20^, ^21^ ^]^ However, there is currently no technique which can directly measure calcium uptake noninvasively. Hence, a method to quantify calcium uptake within live cells, animals, and patients would provide valuable information on cardiac viability and insights into pathological and therapeutic mechanisms.^[^ ^5^, ^6^, ^22^ ^]^

Manganese(II) is an essential trace element and a calcium analogue which enters contractile cardiomyocytes through voltage‐gated calcium channels.^[^ ^23^, ^24^, ^25^, ^26^ ^]^ Mn^2+^ is also an MRI contrast agent. Hence, Mn^2+^ levels can be quantified in vivo using MRI to give surrogate measurements of calcium uptake and thus myocardial viability. Several preclinical^[^ ^27^, ^28^, ^29^, ^30^, ^31^ ^]^ and clinical studies^[^ ^32^, ^33^, ^34^ ^]^ have investigated manganese‐enhanced MRI (MEMRI) for measuring myocardial viability. However, competitive binding of Mn^2+^ to Ca^2+^ channels can be cardiotoxic and has limited the use on Mn^2+^ in MRI.^[^ ^35^ ^]^ Chelation of Mn^2+^ can limit toxicity, but also reduces cell uptake. Combining Mn^2+^ with a calcium supplement has shown great promise for preventing cardio‐depression and has led to the development of EVP1001‐1,^[^ ^28^, ^36^, ^37^ ^]^ a clinical grade contrast agent with great potential for cardiac imaging.

Here, we evaluated in real time the effects of Mn^2+^ with or without the addition of the Ca^2+^ supplement Ca‐gluconate (CaG) on Ca^2+^ transients and action potentials in vitro in mouse and human cardiomyocytes and cardiac contractility in vivo in mice. We then tested if quantitative T1 mapping MEMRI could be used as a surrogate measure of Ca^2+^ uptake in AMI and offer a novel in vivo insight into cardiac inotropy. Finally, we used high‐resolution T1‐weighted MRI to compare MEMRI measurements of infarct size at 1 h, 1 and 14 days after occlusion with those acquired using the current gold‐standard technique, LGE‐MRI.

## 2 Results

### 2.1 Effects of Manganese on Cardiac Electrophysiology In Vitro and Function In Vivo

**Figure 1:**
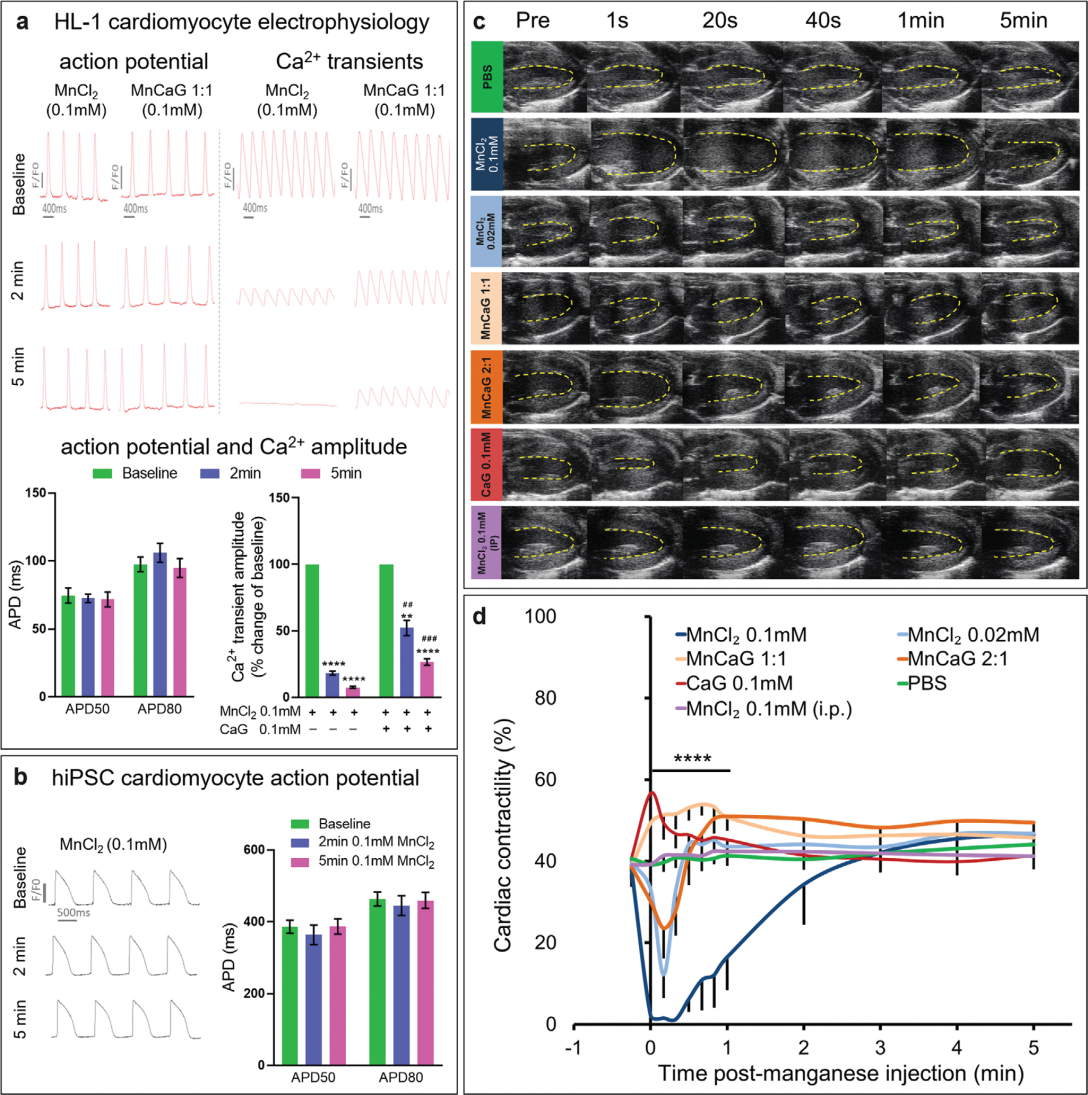
Effects of manganese on cardiac electrophysiology and function: a) Upper panel: Representative optical action potential and Ca^2+^ transient traces from HL‐1 cardiomyocytes at baseline and at 2 and 5 min after superfusion with 0.1 mM MnCl_2_ with/out CaG supplement. Lower panel: APD50 and APD80 were not affected by 0.1 mM MnCl_2_ (*n* = 5), but Ca^2+^ transient amplitude was reduced by 0.1 mM MnCl_2_ and partially restored by supplement with 0.1 mM CaG (*n* = 4). Data were analyzed by paired two‐tailed Student's *t*‐test to compare with baseline **p = 0.0011 ****p < 0.0001 and unpaired two‐tailed Student's *t*‐test to compare MnCl_2_ to MnCl_2_+CaG^‐ ##^ *p* = 0.0011 ^###^ *p* = 0.0003). b) Representative optical action potential traces from human iPSC derived cardiomyocytes at baseline and at 2 and 5 min after superfusion with 0.1 mM MnCl_2_. APD50 and APD80 were not affected by 0.1 mM MnCl_2_ (*n* = 6). c) Representative end systolic ultrasound images acquired before and after injection of the compounds indicated. d) Time course of myocardial contractility quantified as fractional shortening showed immediate reduction in myocardial contractility after i.v. infusion of 0.1 mM MnCl_2_ (*n* = 6) which can be reversed when Mn^2+^ was supplemented with CaG (MnCaG) at either 1:1 or 2:1 MnCl_2_ to CaG ratio (*n* = 6). Mean value ± standard error mean (SEM). Data were compared between groups at each time point by one‐way ANOVA (****p < 0.0001 at 1 s to 1 min post injection) and compared to baseline in each group using two‐way repeated measures ANOVA followed by Dunnett's post hoc test (Table S1, Supporting Information). Raw data are presented in Table S1 in the Supporting Information.

To investigate the effects of Mn^2+^ and Ca^2+^ supplement on cardiomyocyte beating rate and electrophysiology, in vitro studies were performed using mouse HL‐1 cardiomyocytes and human‐induced pluripotent stem cell (hiPSC) derived cardiomyocytes, with the latter now well recognized as the optimal cell type for preclinical compound screening.^[^ ^38^ ^]^ HL‐1 and hiPSC cardiomyocyte beating rates were unaffected by 0.1 mM MnCl_2_ but significantly reduced when superfused with 1 or 2 mM MnCl_2_, respectively. Supplement of 1 or 2 mM MnCl_2_ with 1 or 2 mM CaG restored beating rate in HL‐1 and hiPSC cardiomyocytes, respectively (Figure S1, Supporting Information). Cardiomyocytes were loaded with voltage‐sensitive dye FluoVolt or Ca^2+^ dye Fluo‐4 AM and, based on the dose defined in the above beating rate experiments, optical mapping was performed during superfusion of 0.1 mM MnCl_2_. No changes in action potential duration (APD) were identified but Ca^2+^ transient amplitude was significantly reduced. However, supplement of 0.1 mM MnCl_2_ with 0.1 mM CaG partially restored Ca^2+^ transient amplitude (**Figure** **1** and Figure S1, Supporting Information).

To quantify real‐time effects of Mn^2+^ on myocardial contractility in vivo, studies were performed in mice using b‐mode ultrasound to measure left ventricular fractional shortening prior to and after administration of 2 µL g^−1^ of the following manganese solutions: 0.1 mM MnCl_2_ i.v. (*n* = 6); 0.02 mM MnCl_2_ i.v. (*n* = 4); 0.1 mM MnCl_2_ + 0.1 mM CaG i.v. [MnCaG1:1] (*n* = 6); 0.1 mM MnCl_2_ + 0.05 mM CaG i.v. [MnCaG2:1] (*n* = 6); 0.1 mM CaG i.v. (*n* = 6); phosphate buffered saline (PBS) i.v. (*n* = 3); or 0.1 mM MnCl_2_ i.p. (*n* = 5). These doses were based upon previous publications^[^ ^39^, ^40^, ^41^ ^]^ and our pilot data in which sufficient MRI contrast was generated without obvious effects on cardiac function or animal recovery, behavior, and survival. Ultrasound showed immediate and transient reduction of myocardial contractility after 0.02 mM and 0.1 mM MnCl_2_ i.v., with contractility severely impaired at the higher dose (Figure 1 and Table S1, Supporting Information). This depression was reduced when 0.1 mM MnCl_2_ was supplemented with 0.05 mM CaG and reversed with 0.1 mM CaG. Administration of CaG i.v. showed a transient increase in contractility, while PBS i.v. and 0.1 mM MnCl_2_ i.p. had no effect on contractility (Figure 1). Heart rate was immediately and transiently reduced after 0.1 mM MnCl_2_ i.v. but not significantly altered by any of the other treatments (Figure S1 and Table S2, Supporting Information).

### 2.2 Time Course of Manganese Uptake in Control Mice

To quantify the kinetics and biodistribution of different Mn‐based contrast agents, mice were serially imaged over 24 h after Mn^2+^ injection using T1 mapping MRI (T1m‐MEMRI) to measure the relaxivity (R1) of myocardium, liver, skeletal muscle, and blood. Four formulations of Mn contrast agent were administered: 1) 0.02 mM MnCl_2_ i.v. (*n* = 4); 2) 0.1 mM MnCl_2_ + 0.1 mM CaG i.v. [MnCaG1:1] (*n* = 6); 3) 0.1 mM MnCl_2_ + 0.05 mM CaG i.v. [MnCaG2:1] (*n* = 6); or 4) 0.1 mM MnCl_2_ i.p. (*n* = 5). I.v. injection of 0.1 mmol kg^−1^ MnCl_2_ was not tested as the data presented above indicated it has a significant effect on heart function.

**Figure 2:**
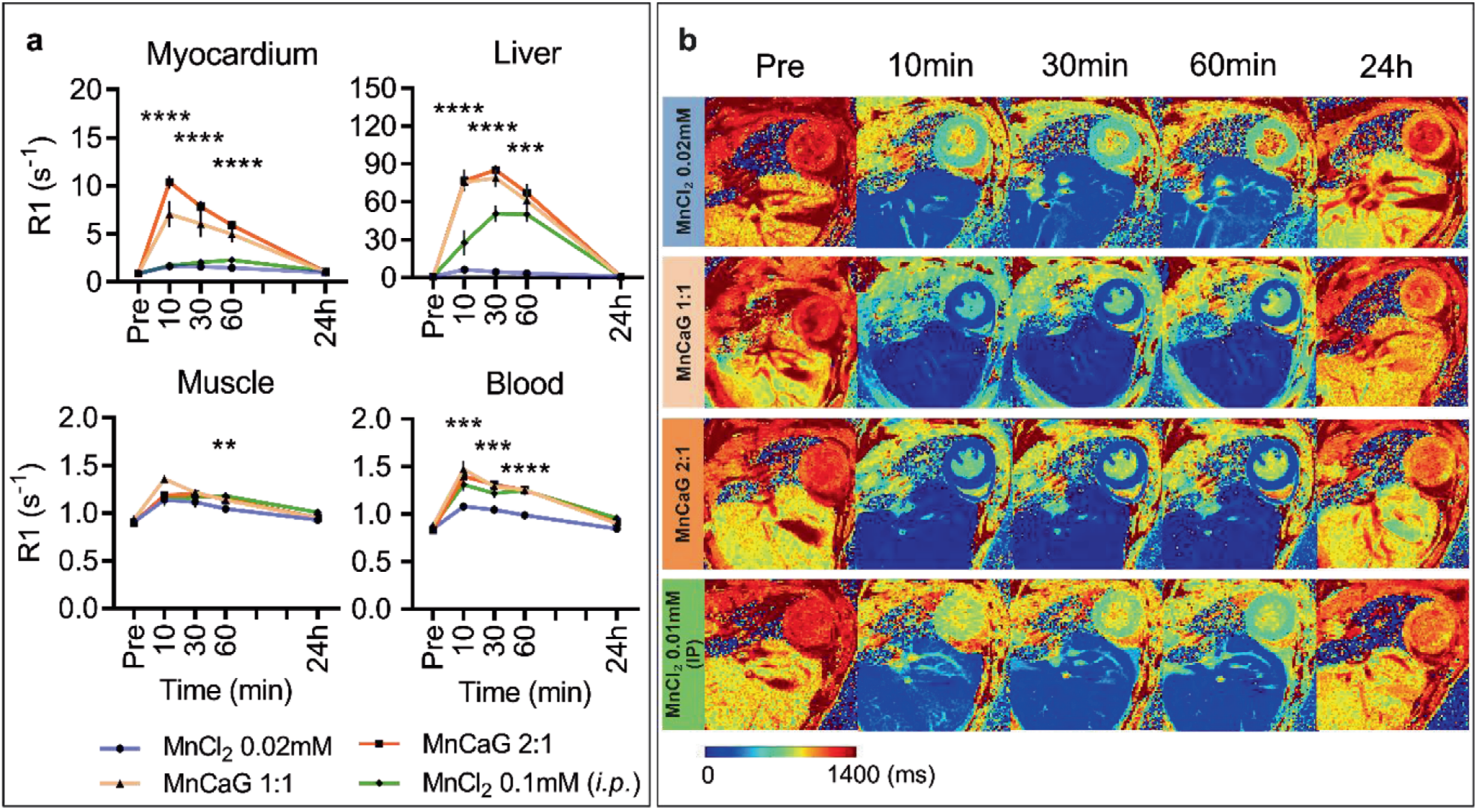
Time course of manganese uptake in control mice. a) R1 values (mean ± SEM) in myocardium, liver, muscle, and blood at baseline, 10, 30, 60 min and 24 h post injection of 1) 0.02 mM MnCl_2_ i.v. (*n* = 6); 2) 0.1 mM MnCl_2_ + 0.1 mM CaG i.v. [MnCaG1:1] (*n* = 5); 3) 0.1 mM MnCl_2_ + 0.05 mM CaG i.v. [mnCaG2:1] (*n* = 5); or 4) 0.1 mM MnCl_2_ i.p. (*n* = 5). Data were analyzed by one‐way ANOVA. Myocardium 10, 30, and 60 min (****p < 0.0001); liver 10, 30 (****p < 0.0001), and 60 min (***p = 0.0002); muscle 60 min (**p < 0.0033); and blood 10 min (***p = 0.0008), 30 min (***p = 0.0005), and 60 min (****p < 0.0001). b) Representative images for each group at baseline, 10, 30, and 60 min and 24 h post injection.

In all groups, myocardial R1 was significantly increased as soon as 10 min after administration. In animals that received i.v. injections, myocardial R1 was greatest at 10 min and reduced from 10 to 60 min, returning to baseline by 24 h (**Figure** **2**). In animals that received i.p. injection, maximal myocardial R1 was delayed to 60 min after injection and remained stable for 3 h owning to the slow, sustained absorption of Mn^2+^ from the peritoneal cavity into the blood. Similar uptake kinetics were observed in the liver, skeletal muscle, and blood.

An estimation of the concentration of Mn^2+^ within the myocardium was made from the in vivo images via a standard curve of MnCl_2_ diluted in serum (Figure S2, Supporting Information). The estimated maximum loading concentration of 0.22 ± 0.03 mM for 0.1 mM MnCl_2_ i.p. is ≈10 times the natural abundance of manganese in the body (≈0.02 mM^[^ ^42^ ^]^) and is equivalent to MnCl_2_ concentrations that showed no effect on in vitro electrophysiology. Therefore, a dose of 0.1 mM MnCl_2_ i.p. was selected for remaining studies given that it had no effect on heart rate or contractility, did not require Ca^2+^ supplement, and gave sufficient and long‐lasting MRI contrast.

### 2.3 T1 Mapping of Manganese Uptake Acutely after MI

As Mn^2+^ offers a functional marker of myocardial viability, we aimed to investigate Mn^2+^ within the myocardium in the first hours after AMI. Mice received i.p. injections of 0.1 mM MnCl_2_ 40 min before permanent coronary occlusion so that the level of Mn^2+^ within the myocardium would be reasonably stable during the imaging time‐points 1 to 3 h after MI. T1m‐MEMRI was performed 1, 2, and 3 h and 2 days after MI. R1 values (1/T1 = relaxivity of the tissue) were analyzed from the area‐at‐risk segments within the territory of the occluded artery (AAR‐MI, *n* = 8) and viable segments remote from the AAR (Remote‐MI, *n* = 8) of infarcted hearts. Naïve mice with the same Mn^2+^ infusion times were used as controls (Viable‐Naïve, *n* = 5).

**Figure 3:**
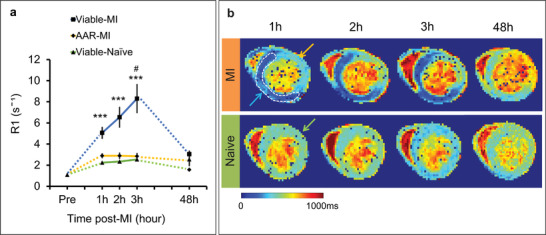
T1 mapping of manganese uptake acutely after MI. a) Time course of changes in R1 over the first 3 h of MI from the area‐at‐risk segments (AAR‐MI, *n* = 8) and viable segments remote from the AAR (Remote‐MI, *n* = 8) of infarcted hearts; and naïve mice with the same Mn^2+^ infusion times (Viable‐Naïve, *n* = 5). Mice received i.p. injections of 0.1 mM MnCl_2_ 40 min before permanent coronary occlusion. Measurements were repeated at 2 days (*n* = 5) after MI (60 min after re‐administration of 0.1 mM MnCl_2_). Statistical significance were calculated using one‐way ANOVA followed by Tukey's post hoc test to compare between groups at 1 h (***p = 0.0004), 2 h (***p = 0.0007), 3 h (***p = 0.0004), and 48 h and using two‐way repeated measures ANOVA followed by Tukey's post hoc test (Viable‐MI group, 1 h versus 3 h, ^#^ *p* = 0.0481). b) Representative T1 maps of infarcted and healthy mice. Blue arrow = Remote‐MI, Yellow arrow = AAR‐MI, Green arrow = Viable‐Naïve.

T1m‐MEMRI revealed that as early as 1 h after MI, R1‐values increased in Remote‐MI tissue compared with AAR‐MI or naïve controls, indicating increased Mn^2+^ uptake in viable myocardium remote from the infarction (**Figure** **3**). R1‐values continued to rise in the Remote‐MI at 2 and 3 h, while no significant changes occurred in the AAR‐MI and Viable‐Naïve groups. At 2 days post‐MI, T1 mapping was repeated 60 min after Mn^2+^ re‐administration in five surviving mice (three mice were euthanized due to severe symptoms of heart disease). R1 values remained higher in the Remote‐MI tissue compared with AAR‐MI tissue. However, Remote‐MI tissue had similar R1 to naïve hearts, while R1 in the infarcted AAR‐MI was lower than controls (Figure 3).

These data show that acutely after ischemic injury, R1 increased (reflecting increased Mn^2+^ uptake) in viable myocytes remote from the AAR, likely due to elevated catecholamine levels acutely post‐MI increasing cardiac work and thus increased Ca^2+^/Mn^2+^ uptake. By 2 days the catecholamine storm had passed^[^ ^43^ ^]^ and R1 levels in the surviving myocardium were normalized, while Mn^2+^ uptake in the AAR was reduced due to irreversible cell death and loss of functional myocytes. These data suggest that MEMRI could be used in preclinical models to optimize novel pharmacological interventions which modulate Ca^2+^ homeostasis acutely after MI, and could be applied in patients to determine efficacy of treatments regulating cardiac inotropy.^[^ ^21^ ^]^

### 2.4 Direct Comparison of Gd‐Enhanced MRI with Mn‐Enhanced MRI in AMI

LGE‐MRI sets the gold‐standard for infarct size quantification in MI. However, accumulation of Gd‐DTPA in the infarct tissue is nonspecific and a contrast agent that actively targets viable myocardium may offer advantages over LGE. We hypothesized that MEMRI could quantify final infarct size earlier than LGE‐MRI and tested this by applying both methods to mice at 1 h, 1 day, and 14 days after MI.

MI was induced by permanent coronary occlusion in 14 mice, which were then randomly assigned to undergo either inversion‐recovery multi‐slice T1‐weighted MEMRI (*n* = 7, 0.1 mmol kg^−1^ MnCl_2_ i.p.) or inversion‐recovery multi‐slice T1‐weighted LGE‐MRI (*n* = 7, 0.6 mmol kg^−1^ Gd‐DTPA i.p.) at 1 h post MI. All 14 animals then underwent both MEMRI and LGE‐MRI again at 1 and 14 days post MI with a contrast washout period of at least 5 h between scans, which we and others^[^ ^37^ ^]^ have shown allows for sufficient Gd‐DTPA washout (Figure S3, Supporting Information).

**Figure 4:**
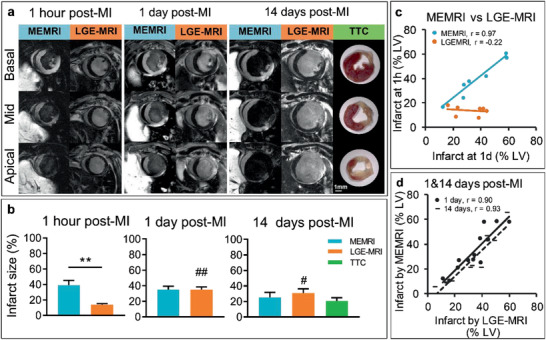
Direct comparison of MEMRI with LGE‐MRI in AMI. a) Representative MEMRI and LGE‐MRI images acquired in mice at 1 h, 1 and 14 days after MI and compared with 14 days histological TTC staining for delineation of infarct area. b) Mean of infarct size measured by MEMRI and LGE‐MRI at 1 h (*n* = 7), 1 day (*n* = 14), and 14 days (*n* = 10) after MI and compared with histological TTC staining. Data were analyzed using unpaired (MEMRI vs LGE‐MRI at 1 h, **p = 0.0015) and paired (MEMRI vs LGE‐MRI at 1 day and 14 days) two‐tailed *t*‐tests and one‐way ANOVA followed by Tukey's post hoc test (LGE‐MRI, 1 h vs 1 day, ^##^ *p* = 0.0083 and 1 h vs 14 days, ^#^ *p* = 0.024). c) Correlation between 1 h versus 1 day MEMRI (*p* = 0.0003) and LGE‐MRI (ns) measurements of infarct size. d) Correlation between MEMRI versus LGE‐MRI measurements of infarct size at 1 (*p* = 0.0002) and 14 days post‐MI (*p* = 0.0001). Correlations were calculated using Pearson correlation.

At 1 h post‐MI, viable myocardium remote from the AAR was enhanced in MEMRI, allowing early delineation of the infarct zone as 39.2 ± 6% of the myocardium, whereas only subtle enhancement within the AAR was observed on LGE‐MRI, leading to a significantly lower measure of infarct zone (13.7 ± 1%, *p* = 0.008; **Figure** **4**a,b). At 1 day post MI, the MEMRI measure of infarct size remained constant (34.9 ± 4%) while the LGE‐MRI measurement significantly increased to a level comparable with MEMRI (34.8 ± 4%) (Figure 4a,b). The correlation between 1 h and 1 day MEMRI infarct size was strong (*R* = 0.97, *p* < 0.001), while there was no correlation between 1 h and 1 day LGE‐MRI measurements of infarct size (Figure 4c).

Between 1 and 14 days, four mice (two from each group) were euthanized owing to severe symptoms of heart disease. The ten surviving mice were imaged with both MEMRI and LGE‐MRI at 14 days. The percentage infarct sizes had reduced when measured by either technique (MEMRI 25.4 ± 6%, LGE‐MRI 31.2 ± 6%) owing to the expected hypertrophy of viable myocardium and thinning of the scar. There was a strong correlation between MEMRI and LGE‐MRI measurements of infarct size at both 1 and 14 days (Figure 4d). Both MEMRI and LGE‐MRI indicated similar 14 days infarct sizes to ex vivo infarct quantification using triphenyl‐tetrazolium chloride (TTC) staining (20.9 ± 4%). End diastolic volume, end‐systolic volume, ejection fraction, and left ventricular (LV) mass were similar between the groups that initially underwent MEMRI or LGE‐MRI (Figure S4, Supporting Information). Taken together, these data rule out the possibility that the greater infarct sizes measured by MEMRI at 1 h were owing to variability in surgically induced damage between groups and confirm that manganese enhancement directly reflects acute myocardial viability and offers an earlier indicator of final infarct size than LGE‐MRI.

## 3 Discussion

In this study, we used real‐time optical mapping and ultrasound imaging to demonstrate that the cardio‐depressant effects of Mn^2+^ can be negated if administered with a calcium supplement or injected i.p. to permit slow release. These approaches yielded sufficient Mn^2+^ uptake within the viable myocardium of mice to generate contrast enhancement in MRI and were then used to evaluate Ca^2+^ homeostasis and infarct size in the first hours after MI.

The clinical use of manganese‐enhanced MRI has been limited as it is well documented that high doses of i.v. Mn^2+^ can reduce myocardial contractility.^[^ ^42^ ^]^ A dose >5 mg kg^−1^ (0.04 mM) MnCl_2_ causes decreased heart rate and blood pressure in dogs^[^ ^44^ ^]^ and LD_50_ is 0.3 mM in rats.^[^ ^45^ ^]^ However, low dose MEMRI has been safely tested in healthy human volunteers via a 3 min infusion of MnCl_2_ to a total of 5 µM.^[^ ^34^ ^]^ Chelation can dramatically reduce toxicity, with MnDPDP having an LD_50_ of 1.89 mM,^[^ ^45^ ^]^ and has been tested in humans at 15 µM infused over 30 min.^[^ ^32^, ^33^ ^]^ However, myocardial uptake and T1 shortening is also reduced several fold as the chelated form does not pass through calcium channels,^[^ ^27^, ^45^ ^]^ meaning a greater dose is needed to provide sufficient unbound Mn^2+^ to generate adequate signal enhancement. It has been shown that CaG‐based EVP‐1001‐1 provides similar T1 shortening to MnCl_2_,^[^ ^27^ ^]^ thus offering a potent agent for imaging myocardial viability. Given the renewed clinical interest in using Mn^2+^ as a functional contrast agent, it is important to gain additional in vitro and preclinical data on its safety and in vivo applications. To our knowledge, this is the first study to investigate the effects of different Mn^2+^ formulations on action potential duration, Ca^2+^ transients, real‐time in vivo cardiac contractility, and MnCl_2_ uptake.

Action potentials and Ca^2+^ handling play pivotal roles in coordinating cardiac contraction. Alterations in action potential duration, Ca^2+^ transients, and contractility are associated with many forms of heart disease; are important targets for several antiarrhythmic treatments; and thus, need to be considered when administering substances such as functional contrast agents. We observed no changes in action potential duration or beating rate when HL‐1 or hiPSC cardiomyocytes were superfused with 0.1 mM MnCl_2_, but beating rate was reduced by 1 or 2 mM MnCl_2_, respectively, with rapid recovery upon washout. Conversely, in vivo measurements of cardiac contractility made using real‐time ultrasound showed that i.v. infusion of 0.02 and 0.1 mM MnCl_2_ rapidly and transiently reduced myocardial contractility, with 0.1 mM MnCl_2_ infusion also lowering heart rate. The greater effects observed in vivo, despite lower MnCl_2_ infusion doses are due to the difference in myocyte MnCl_2_ loading between the in vitro and in vivo conditions. To account for this, we performed in vivo quantification of MnCl_2_ mediated changes in myocardial T1, then via a standard curve calculated that i.v. infusion of 0.02 mM MnCl_2_ results in a myocardial loading of 0.24 mM MnCl_2_ at 10 min after injection, while i.v. infusion or i.p. injection of 0.1 mM MnCl_2_ resulted in >1.5 mM and 0.22 mM MnCl_2_ myocardial loading, respectively. This indicates that true MnCl_2_ myocardial loading in our in vivo experiments is similar to the MnCl_2_ concentrations used in vitro (1 mM and 0.1 mM), allowing us to extrapolate findings between the two conditions. It is also important to consider that while cells in culture are exposed to a constant and well‐defined MnCl_2_ concentration, during in vivo infusion the concentration of the i.v. MnCl_2_ bolus when it first passes the heart will be several fold higher than when at equilibrium. This initial spike in Mn^2+^ is responsible for the immediate and transient effect observed with i.v. infusion of 0.02 mM MnCl_2_, which is absent after i.p. injection and in vitro, despite a similar Mn^2+^ concentration (0.1 mM to 0.2 mM) at equilibrium. Additionally, the vasodilatory effects of MnCl_2_ may play an important role in the changes observed in vivo through reduction in afterload, although this mechanism was not evaluated here.

Ca^2+^ transient amplitude was reduced by 0.1 mM MnCl_2_. Reduction in Ca^2+^ transient amplitude has previously been shown to detect Mn^2+^ entry in the cytoplasm^[^ ^46^ ^]^ due to the ability of Mn^2+^ to quench calcium‐sensitive dyes^[^ ^47^ ^]^ and thus reduce Ca^2+^ mediated Fluo‐4 AM fluorescence. Hence, our data cannot determine if Mn^2+^ affects Ca^2+^ transients, but does confirm that Mn^2+^ was entering the cardiomyocytes. However, both our in vitro and in vivo data suggest that Ca^2+^ uptake by cardiomyocytes is not affected by 0.1 mM MnCl_2_ as no effects on HL‐1 or hiPSC cardiomyocyte electrophysiology or heart contractility were observed when the administered dose reached equilibrium. As previously shown in rat hearts, such low concentrations of Mn^2+^ do not reduce Ca^2+^ entry via slow Ca^2+^ channels.^[^ ^48^ ^]^ Once Mn^2+^ concentration approaches that of Ca^2+^, the higher affinity of channel binding sites for Mn^2+^ than Ca^2+[^ ^35^, ^49^ ^]^ results in a reduction of Ca^2+^ entry and dysfunction. This is supported by the significant reduction of HL‐1 and hiPSC cardiomyocyte beating rate, in vivo contractility, and heart rate observed in our studies at high doses of MnCl_2_, together with previously reported findings in Langendorff perfused hearts and in vivo in several species.^[^ ^24^, ^26^, ^33^, ^34^, ^35^, ^39^, ^42^, ^44^, ^45^, ^50^, ^51^ ^]^

Co‐administration of Mn^2+^ with Ca^2+^ supplements has been proposed as a strategy to prevent the above effects.^[^ ^28^, ^36^, ^37^ ^]^ Indeed, supplementing MnCl_2_ with CaG prevented changes in cardiomyocytes beating rate in vitro. Similarly, supplementing 0.1 mM MnCl_2_ with 0.1 mM CaG prevented the reduction of in vivo cardiac contractility and heart rate induced by 0.1 mM MnCl_2_ alone. However, there are drawbacks of Ca^2+^ supplementation. First, elevation of extracellular Ca^2+^ may impair Mn^2+^ influx into viable myocytes as demonstrated by the partial restoration of Ca^2+^ transient amplitude in vitro after supplementation with CaG (Figure 1a). This led to reduced contrast enhancement observed in vivo after CaG supplement (Figure S2, Supporting Information). Second, Ca^2+^ may induce a positive inotropic response as observed when 0.1 mM CaG or a 1:1 ratio of 0.1 mM Mn^2+^:CaG was infused (Figure 1d). Finally, supplementation with high concentrations of Ca^2+^ may promote side effects such as arrhythmias, although none were observed in this study. Together, these data suggest 0.1 mM MnCl_2_ is unlikely to be arrhythmogenic or cardio depressive, and that loading the myocardium by slow MnCl_2_ infusion rather than a rapid bolus, or in combination with a mild Ca^2+^ supplement, can facilitate the safe use of manganese‐enhanced MRI in preclinical studies. The long‐term toxicity of manganese exposure has been investigated in numerous publications (reviewed in refs. ^[^ ^6^, ^42^ ^]^), which indicate that a single injection of a low Mn^2+^ dose is unlikely to cause lasting adverse events. Furthermore, although our studies were focused on the acute effects of Mn^2+^ administration, it is important to note that all mice survived even higher dose protocols; weight loss was not observed; behavior was normal; and no unexpected events or deaths were reported in any of the control mice that received Mn^2+^.

T1 mapping MRI was used to quantify the kinetics and biodistribution of different Mn‐based contrast agents over 24 h. All Mn^2+^ doses increased R1 within 10 min of administration to a level sufficient to induce image contrast. Similar uptake kinetics were observed in the heart, liver, skeletal muscle, and blood. As expected liver uptake was extremely high, reflecting the clearance pathway for Mn^2+^;^[^ ^42^ ^]^ skeletal muscle uptake was low because no muscle contraction occurred while mice were anesthetized; and Mn^2+^ levels in blood were low because of the short blood half‐life of Mn^2+^.^[^ ^50^ ^]^ In the heart, intravenous administration of the higher ratio of CaG to MnCl_2_ (1:1) did not increased R1 to the same extent as when the lower CaG dose was administered (MnCl_2_ 2:1 CaG). This difference was not observed in the R1 of the liver because liver uptake does not predominantly occur via calcium channels.^[^ ^42^ ^]^ These data, together with the partial restoration of Ca^2+^ signal observed in our optical mapping studies when CaG supplement was provided, add further evidence that there is active uptake of Mn^2+^ into contractile cells via Ca^2+^ channels.

We next evaluated Ca^2+^ homeostasis acutely after permanent coronary occlusion using T1‐mapping of Mn^2+^‐mediated changes in R1. This is the first study to quantify Mn^2+^ uptake in the first hours of AMI using T1‐mapping. Previous studies have used T1‐weighted imaging to investigate Mn^2+^ uptake,^[^ ^37^, ^39^, ^41^, ^52^, ^53^ ^]^ but these approaches do not quantify R1, so are only sensitive to relative changes in contrast rather than direct measurements of Mn^2+^ uptake. We had initially hypothesized that the Mn^2+^ preloaded myocardium would lose contrast agent in the AAR over the time‐course of cell death. Had we just used data from T1‐weighted imaging, then this theory may have appeared true, as we did see a relative change in contrast between the AAR and the viable remote myocardium. However, quantitative T1‐mapping allowed us to determine that the reason for the difference was continued Mn^2+^ uptake in viable myocardium rather than loss from the ischemic zone. This has important implications for how T1m‐MEMRI could be used for evaluating the efficacy and dosing of inotropic drugs in AMI as Mn^2+^ levels may directly reflect the impact of pharmacological intervention on cardiac inotropy. The effects of positive inotropic drug dobutamine and calcium channel blocker diltiazem can be detected using T1‐weighted MEMRI.^[^ ^40^ ^]^ Hence, the use of quantitative T1m‐MEMRI may further aid clinical management in acute and chronic MI.^[^ ^21^, ^29^ ^]^

Finally, we compared the ability of Gd‐DTPA and Mn^2+^‐enhanced T1‐weighted MRI to delineate the area of infarction 1 h after coronary occlusion, then followed up the same mice at 1 and 14 days. At 1 h, infarct sizes as assessed by LGE‐MRI were significantly lower than MEMRI, and while the MEMRI measurements remained stable over 24 h, the LGE‐MRI measurements increased to a similar level to that reported by MEMRI. It is not possible to combine Gd‐DTPA and Mn^2+^‐enhanced MRI at the same time as both act through the same NMR process of increasing R1 relaxivity. This made it impossible to perform a direct comparison in the same mice at 1 h after infarction. However, similar to previous reports,^[^ ^37^ ^]^ we showed that >4 h of Gd‐DTPA washout was sufficient to permit subsequent MEMRI, meaning all mice could be scanned at 22–27 h post infarction using both methods. These results, along with standard measures of cardiac structure, ejection fraction, and infarct size, revealed that the different groups of mice sustained equivalent myocardial injuries which resulted in similar cardiac impairment at later time points, confirming for the first time that MEMRI provides an early biomarker on final infarct size after permanent coronary occlusion.

Previous studies have demonstrated the accuracy of MEMRI in delineating infarct size in experimental MI^[^ ^37^, ^39^, ^41^, ^52^, ^53^ ^]^ and its usefulness in interrogating pathophysiology,^[^ ^30^, ^54^, ^55^ ^]^ but none have investigated such early changes after coronary occlusion in comparison to LGE‐MRI. Our data suggest that myocytes within the AAR rapidly stop internalizing Mn^2+^ under ischemic conditions, while our T1 mapping results show increased Mn^2+^ uptake in the viable myocardium remote from the infarct. These processes combine to rapidly produce high contrast between viable and ischemic myocardium allowing early delineation of the region of occlusion. The membrane rupture and edema that underlie Gd‐DTPA accumulation in LGE‐MRI occur later in the pathological process, meaning LGE‐MRI underestimates the final infarct size during the first hours post MI, when cell death is incomplete. Several studies have shown that LGE‐MRI measurements of infarct size evolve with time after MI,^[^ ^56^, ^57^, ^58^, ^59^ ^]^ but none have made measurements as early as 1 h after coronary occlusion. It is of interest that we observed patchy mid‐wall Gd‐DTPA enhancement in all mice at 1 h. LGE‐MRI is typically performed several hours after MI once cell death has occurred. Our data indicate that small regions of LGE are present soon after MI and could represent irreversible cell death in the most severe region of myocardial ischemia.

It has been proposed that the mismatch between infarct measurements made using MEMRI and LGE‐MRI could represent the viable infarct border zone of the ischemic region.^[^ ^37^ ^]^ This is an interesting phenomenon to investigate further, but as our study used a permanent coronary occlusion model, this border zone would be minimal and was not identified when images were coregistered.

The ability to noninvasively quantify myocardial viability could have important diagnostic and prognostic applications across a range of cardiac diseases. First, accurate identification of viable myocardium could be used to identify which patients would benefit from percutaneous intervention or coronary artery bypass grafting.^[^ ^60^ ^]^ Recent clinical trials have cast doubt on the value of PET‐ and SPECT‐based myocardial viability imaging,^[^ ^7^, ^8^ ^]^ thus increasing the need for more accurate methods. Additionally, MEMRI could have a role to play in phenotyping hypertrophic and dilated cardiomyopathies, and improving diagnosis of stress “takotsubo” cardiomyopathy.^[^ ^61^ ^]^ There is also great potential for evaluation of routine and experimental therapies including optimization of pharmacological doses to regulate cardiac inotropy;^[^ ^21^ ^]^ monitoring success of regenerative therapies;^[^ ^62^ ^]^ and interrogating viability of engineered heart tissues.^[^ ^63^, ^64^, ^65^ ^]^

MEMRI is the only noninvasive imaging technique currently able to act as a surrogate measure of calcium uptake in vivo. Optical methods can measure calcium‐sensitive dyes in vitro, but have limitations for in vivo applications as they require administration of toxic agents/genetic modifications, invasive surgery, implantation of optical windows, and are only able to monitor calcium near the surface of peripheral organs.^[^ ^66^ ^]^ Radioisotopes of manganese for positron emission tomography do exist and have recently been shown to detect changes in functional beta‐cell mass in mouse models of diabetes.^[^ ^67^, ^68^ ^]^ However, radioisotopes of manganese have not been investigated for monitoring cardiac viability and few centers have cyclotrons which can synthesize these isotopes, currently making them impractical for clinical use.

There are disadvantages to using MEMRI. Although manganese‐based contrast agents have been approved for human use,^[^ ^5^, ^32^ ^]^ current access to clinical grade agents is limited. However, given the low cost of MnCl_2_, its long storage life, and ease of distribution, it will be possible to increase the availability of manganese‐based agents once their clinical usefulness is highlighted by studies such as the one presented here. As manganese generates contrast through the same T1 shorten mechanisms as gadolinium‐based contrast agents, it does mean routine LGE‐MRI could not easily be acquired at exactly the same time as MEMRI. However, we and others^[^ ^28^ ^]^ have shown that delays between contrast administration can overcome this problem. Additionally, MR pulse sequences used for LGE‐MRI can also be used for MEMRI, meaning the acquisition protocols are already available within cardiovascular MRI centers worldwide.

## 4 Conclusions

Our data indicate that manganese‐enhanced MRI offers an important new method for evaluating myocardial viability within the first hour of MI. High doses of Mn^2+^ reduced Ca^2+^ transients and myocardial contractility but did not alter action potential. Supplement with CaG reduced these effects, supporting the use of CaG in Mn^2+^‐based MRI contrast agents. MEMRI showed that R1 increases acutely after ischemic injury (reflecting increased Mn^2+^ uptake) in viable myocytes, suggesting that MEMRI could be used to optimize novel pharmacological interventions that modulate Ca^2+^ homeostasis acutely after MI. Finally, we showed that MEMRI could quantify infarct size earlier than LGE‐MRI and provides a sensitive early measure of AAR before irreversible cell death occurred. This method will be of great use in the preclinical evaluation of treatments that target events early in ischemic injury and reperfusion, and may offer a new biomarker for evaluating the success of reperfusion therapy. The re‐emergence of safe, clinical grade Mn^2+^‐based contrast agents opens the possibility of direct evaluation of myocardial viability early after ischemic onset in patients with AMI.

## 5 Experimental Section

##### In Vitro Study

HL‐1 cardiomyocytes were cultured on gelatin‐fibronectin‐coated plastic coverslips (13 mm, Sarstedt) at a density of 160 000 cells per coverslip in Claycomb medium supplemented with 10% fetal bovine serum, 2 mM L‐glutamine, 0.1 mM norepinephrine, and 1% penicillin–streptomycin (all Sigma). hiPSC cardiomyocytes (iCell cardiomyocytes2, Cellular Dynamics International) were cultured on fibronectin‐coated 10 mm glass bottom dishes (MatTek) at a density of 60 000 cells per dish in maintenance medium (Cellular Dynamics) for 7 days prior to functional assessments. Cells were loaded with voltage‐sensitive dye FluoVolt (1:1000 dilution in serum‐free medium, 25 min at 37 °C 5% CO_2_) or Ca^2+^ dye Fluo‐4AM (4 µM plus 1 mM Probenecid for 20 min followed by 20 min de‐esterification). Optical mapping was performed with the Photometrics Evolve 512 EMCCD camera (Photometrics, Tucson, AZ) mounted on a custom macroscope (Cairn Research) equipped with a water‐immersion 20x objective, appropriate excitation/emission filters, and 470 nm light‐emitting diode illumination, using the recording software WinFluor. Action potential recordings were performed at 1000 fps, from a 295 × 295 µm area; Ca^2+^ transient recordings were performed at 200 fps, from an 820 × 820 µm area. Cardiomyocytes were superfused with Normal Tyorde's solution (140 mM NaCl, 4.5 mM KCl, 10 mM glucose, 10 mM 4‐(2‐hydroxyethyl)‐1‐piperazineethanesulfonic acid, 1 mM MgCl_2_, 1.8 mM CaCl_2_, pH 7.4) containing 0.1 mM MnCl_2,_ or 0.1 mM MnCl_2_ supplemented with 0.1 mM CaG in 1 to 1 ratio, at 36 °C. APD and Ca^2+^ transient amplitude were analyzed using OPTIQ (Cairn Research). Optical mapping signals were filtered using a Gaussian spatial filter (radius 2 pixels) before relevant parameters were extracted. APD was measured as the time from the upstroke to 50% and 80% repolarization (APD50 and APD80). Ca^2+^ transient amplitude was measured as peak fluorescence amplitude relative to background fluorescence and presented as % change of baseline, for each of the five regions of interest selected in each dish (*n* = 4 independent biological replicates). For beating rate assessment, HL‐1 cardiomyocytes were superfused with Normal Tyorde's solution containing increasing concentration of MnCl_2_ (0–1 mM; 5 min each), or MnCl_2_ (0–1 mM) supplemented with CaG in 1 to 1 ratio. Beating rate was quantified by measuring the time between ten consecutive action potential peaks, and dividing 60 s by the average value to obtain the number of beats per minute. For hiPSC cardiomyocytes beating rate assessment, spontaneous contractions were recorded using a brightfield microscope. For each monolayer, three different regions were recorded at 60 fps for ≈30 s. Beating rate was defined as number of contractions/recording time. Values were reported as % change relative to baseline at each specific time point to take into account possible time‐dependent variability in beating rate.

##### Study Approval for Animal Work

All protocols were approved by the University College London Biological Services Ethical Review Committee and licensed under the UK Home Office regulations and the Guidance for the Operation of Animals (Scientific Procedures) Act 1986 (Home Office, London, United Kingdom). All experiments were performed following institutional ethical guidelines and regulations and in accordance with the ARRIVE guidelines. Group sizes were determined using power calculations based on previous experimental data using these specific cell types and animals models. Animals were randomized to groups based on the randomization tool in the NC3Rs Experimental Design Assistant.

##### Mouse Model of MI

Induction of MI was performed using a protocol previously established in the laboratory.^[^ ^69^ ^]^ 11 ± 1 weeks old C57Bl/6 mice were subjected to MI by ligation of the left anterior descending (LAD) coronary artery. Briefly, general anesthesia was induced and maintained in the mice using isoflurane at 4% and 1.5%, respectively, in 1.5 L min^−1^ O_2_. Analgesia was provided using a 0.1 mg kg^−1^ subcutaneous injection of buprenorphine at the beginning of surgery, and both 6 and 24 h post‐surgery. Scoring sheets for adverse events were employed daily. Mice were positioned supine on a heated operating table to maintain physiological body temperature, which was monitored using a rectal thermometer. Artificial respiration was provided by a ventilator (MiniVent Type 845, Harvard Apparatus) via oral intubation. Open‐chest surgery was performed to gain access to the heart. The LAD was identified, and a suture was placed ≈2 mm below the tip of the left atrium. Occlusion of the LAD artery resulted in an immediate blanching of the anterior wall of the left ventricle indicative of myocardial ischemia. The ligature was left in place, all surgical openings were closed, and the animal was recovered. Surgeries were conducted in a controlled, sterile surgical suite with aseptic operating practices.

##### Contrast Agents

Manganese (II) chloride, 1.00 ± 0.01 m solution (Sigma Chemical Co., St. Louis, MO) was used for the echocardiography study and manganese‐enhanced MRI experiments. MnCl_2_ was diluted in NaCl 0.9% solution to achieve the desired concentrations. Calcium‐gluconate (Sigma Chemical Co., St. Louis, MO) was used as a calcium supplement to manganese. Calcium‐gluconate powder was dissolved in NaCl 0.9% solution to obtain a stock solution and diluted to achieve the desired concentrations. Six different solutions were prepared: manganese (II) chloride at 50 and 10 mM concentration, calcium‐gluconate at 50 mM concentration, manganese with calcium supplement (calcium‐gluconate) in 1:1 and 2:1 ratio, and PBS. Manganese was supplemented with calcium while maintaining the same number of manganese ions in the solution. Two ratios of manganese‐calcium contrast agents were used: 50 mM MnCl_2_ was supplemented with 50 mM calcium‐gluconate to produce 1:1 ratio and 50 mM MnCl_2_ supplemented with 25 mM calcium‐gluconate to produce 2:1 ratio. PBS was used as the control group. All solutions were administered with a volume of injection of 2 µL g^−1^ body weight via intravenous injections and intraperitoneal injection. Gadolinium‐diethylenetriamine pentaacetic acid‐bismethylamide (Gd‐DTPA‐BMA – Omniscan, GE Healthcare, Hatfield, United Kingdom) was used to study to directly compare Gd‐enhanced MRI with Mn‐enhanced MRI in AMI. Mice were randomly assigned to groups.

##### Echocardiography

Assessment of in vivo cardiac contractility was performed as described previously^[^ ^3^, ^62^ ^]^ using a Vevo 2100 (FUJIFILM Visualsonics, CA, USA) system with 550D 30 MHz transducer. Mice were anesthetized with 1.5–2.0% isoflurane in 2 L min^−1^ O_2_ and positioned supine on a physiological monitoring platform which simultaneously regulated body temperature and measured respiratory and electrocardiogram traces. A rectal probe was inserted to monitor the temperature of the animal during the imaging session. Any changes in cardiac physiology were recorded throughout the experiments. The mouse tail vein was cannulated for administration of Mn‐based contrast agents. Prior to imaging, hair removal cream was applied to the chest to reduce attenuation of the ultrasound signal. Real‐time ultrasound images were acquired at baseline and immediately post‐injection of several Mn‐based contrast agents. To maintain the same anatomical positioning and to be able to acquire images at every 10 s, ultrasound images were acquired only in the parasternal long axis view at baseline and throughout the imaging up to 5 min post‐injection. LV fractional shortening was measured from the mid‐left ventricle of B‐mode acquisitions.

##### MRI—Manganese‐Phantom Study

A phantom study was conducted for quantification of estimated Mn^2+^ concentration in the myocardium using different manganese‐based contrast agents formulations. MnCl_2_ solutions with concentrations of 0, 50, 100, 150, 200, 250, 300, 350, 400, 500, 550, and 1000 µM were made up in 0.9% NaCl in Eppendorf tubes. Some phantoms were made up in serum, to make the measurements more biologically relevant, while some were made in a ratio of 1:1 and 2:1 with calcium‐gluconate. All phantoms were scanned using quantitative T1 mapping in which the imaging parameters were chosen to match the imaging parameter used in the in vivo study to enable a direct comparison of the results to give an idea on Mn^2+^ content in the heart.

##### MRI—In Vivo T1‐Mapping MRI

Cardiac T1 Mapping was performed using a Lock‐Locker (LL) Inversion Recovery sequence in a single mid‐ventricular short axis slice as described previously^[^ ^3^, ^70^ ^]^ (TE = 0.99 ms, TRir = ≈4 s, TI = ≈100–1800 ms (depending on the heart rate), FOV = 25.6 × 25.6 mm, matrix size = 128 × 128, flip angle = 20°, and slice thickness = 1.5 mm). Typical values for these parameters result in an acquisition time around ≈8 min for single slice depending on the heart rate. T1 maps were generated by performing pixel‐wise curve fitting as described above using in house Matlab code (Mathworks, Inc., Natick, USA) based on the Nelder‐Mead simplex method.^[^ ^70^ ^]^ The myocardium, blood pool regions, liver, and muscles T1 were segmented from T1 maps to calculate the mean T1 value. The relaxation rate, the R1 value, is calculated as R1 = 1/T1.

##### MRI—In Vivo T1 Mapping of Manganese and Gadolinium Washout

So that LGE and MEMRI could be performed in the same animal, a pilot study was first undertaken in separate animals to assess clearance of gadolinium and manganese. T1 mapping was performed in eight mice at 7 days after infarction prior to contrast injection and following 0.6 or 0.1 mmol kg^−1^ i.p.* injection* of Gd‐DTPA or MnCl_2_, respectively. Imaging of a single slice was repeated at seven time points (baseline, 30 min, 1, 2, 4, 5, and 24 h) to assess the changes in R1 values (1/T1) reflecting the amount of contrast in the infarcted area, remote myocardium, and blood pool region (Figure S3, Supporting Information).

##### MRI—In Vivo T1‐Weighted LGE‐MRI and MEMRI

Imaging was performed as described previously.^[^ ^3^, ^71^ ^]^ In brief, Gd‐DTPA and MnCl_2_ were administered via intraperitoneal injection as a single bolus of 0.6 or 0.1 mmol kg^−1^, respectively. Mice were randomly assigned to receive either Gd‐DTPA or MnCl_2_ soon after infarction for the 1 h scans. For later time points, late gadolinium enhancement MRI (LGE‐MRI) was performed first in Figure S3 in the Supporting Information with a wait of at least 5 h, before injecting manganese to performed manganese‐enhanced MRI (MEMRI) to ensure the independence of contrast‐enhancement patterns. The longer washout time for MnCl_2_ prevented it being administered first. Data also showed that, LGE‐MRI was best performed within 20 min post‐Gd injection and 60 min post‐Mn injection for MEMRI. Images were acquired using a multi‐slice IR‐GRE (IRmSL) sequence with a single inversion time (TI) point and flip angle of 90°. The imaging sequence was used as described previously.^[^ ^70^ ^]^ The optimum TI was selected from the multiple frames of the LL inversion recovery sequence performed prior to the IRmSL sequence. TI was selected to null the remote myocardium for LGE‐MRI, while for MEMRI, TI was selected to null the infarcted myocardium. This provided the best contrast between healthy and infarcted myocardium. The repetition time between each slice pulse (sTR) was 3 ms, with a sequential acquisition order typically, eight to ten short‐axis slices to cover the entire heart with the following parameters: TE = 3.04, TR = 1.11 ms, flip angle = 90°, slice thickness = 1.0 mm, FOV = 25.6 × 25.6 mm, and matrix size = 128 × 128.

LGE‐MRI and MEMRI images were analyzed using ImageJ software (National Institutes of Health) with semiautomated LV mass and infarct mass. LV mass was measured by semiautomated segmentation of the myocardial area in all slices of the inversion recovery acquisition. LGE‐MRI enhanced areas (hyperintense region) and MEMRI defect areas (hypointense region) were designated infarct areas. The extent of defect areas or enhanced areas was defined as any tissue with image magnitude (signal intensity, SI) above (LGE‐MRI) and below (MEMRI) a pre‐defined threshold as compared to the SI of remote myocardium. Infarct mass measurement was performed in every slice by visual assessment and manual tracing of the enhanced area in LGE‐MRI and MEMRI. The LV and infarcted mass are calculated as segmented LV and infarcted area multiplied by the slice thickness (1 mm) and the specific gravity of the myocardium (1.05). Percent of infarct mass was calculated as infarct mass/total LV mass.

##### MRI—CINE MRI

Cine imaging was acquired in the short axis view using a stack of eight to ten slices to assess cardiac function. A cardiac and respiratory‐gated gradient echo sequence was used to acquire the cine images. Imaging parameters were as follows: TE = 1.18 ms, TR = 5 ms, flip angle = 15°, slice thickness = 1 mm, FOV = 25.6 × 25.6 mm, matrix size = 128 × 128, number of signal averages = 2. Analysis of cardiac function from CINE images was performed using the freely available software Segment (Medviso, Lund, Sweden). The software package applied an automatic segmentation algorithm to the images, which could also be corrected manually. Segmentation of the LV endocardium in all end‐diastole slices gave end diastolic volume (EDV). Similarly, endocardial segmentation in all end‐systole slices gave end systolic volume (ESV). Stroke volume (SV) and ejection fraction (EF) were calculated from the EDV and ESV.

##### Histological Staining to Quantify MI Size

In order to validate cardiac MRI measurements of infarct size, hearts were subjected to ex vivo histological staining using TTC for determining MI size. Briefly, immediately following completion of CMR, mice were sacrificed, the chest was rapidly opened, the femoral vessel was cut, and 5 mL of high potassium (50 mM) PBS was slowly infused via the LV cavity to arrest the heart at diastole. The heart was then rapidly excised, frozen, and then sliced in six to eight parallel short‐axis sections. The heart sections were immersed in freshly prepared TTC (1% TTC in PBS, pre‐warmed to 37 °C) for 15 min. The samples were next placed in formalin for a maximum of 90 min to enhance the red/pale contrast between viable and necrotic tissue. Infarct sizes were manually measured using ImageJ software and compared with the corresponding LGE‐MRI and MEMRI images.

##### Statistical Analysis

All statistical analyses were performed using GraphPad Prism software Version 8.0. All data were presented as mean ± SEM. Differences were considered significant when *p* < 0.05. Comparative group *p* values were determined via Student's *t*‐test, one‐way analysis of variance (ANOVA), and two‐way ANOVA followed by Tukey's post hoc test or Dunnett's post hoc test for multiple‐group comparisons.

The statistical significance in Figure 1a (*n* = 5, 0.1 mM MnCl_2_; n = 4, MnCl_2_^+^ CaG^+^) and Figure 1b (*n* = 6) was calculated using paired (for comparison with baseline) and unpaired (for comparison between groups) two‐tailed Student's *t*‐tests. Data in Figure 1d (*n* = 6, 0.1 mM MnCl_2_ i.v.; *n* = 4, 0.02 mM MnCl_2_ i.v.; *n* = 6, 0.1 mM MnCl_2_ + 0.1 mM CaG i.v.; *n* = 6, 0.1 mM MnCl_2_ + 0.05 mM CaG i.v.; *n* = 6, 0.1 mM CaG i.v.; *n* = 3, PBS i.v.; and *n* = 5, 0.1 mM MnCl_2_ i.p.) were analyzed using one‐way ANOVA (compared between groups at each time points) and using two‐way repeated measures ANOVA followed by Dunnett's post hoc test (Table S1, Supporting Information).

The statistical significance in Figure 2a (*n* = 6, 0.02 mM MnCl_2_ i.v.; *n* = 5, 0.1 mM MnCl_2_ + 0.1 mM CaG i.v.; *n* = 5, 0.1 mM MnCl_2_ + 0.05 mM CaG i.v.; *n* = 5, 0.1 mM MnCl_2_ i.p.) was analyzed using one‐way ANOVA.

Data analysis of Figure 3a (Viable‐MI, *n* = 8; AAR‐MI, *n* = 8; and Viable‐Naïve, *n* = 5) was performed using one‐way ANOVA followed by Tukey's post hoc test (compared between groups at each time points) and two‐way repeated measures ANOVA with Turkey's post hoc test (compared between time points in each group).

Data in Figure 4b were analyzed using unpaired (MEMRI vs LGE‐MRI at 1 h, *n* = 7) and paired (MEMRI vs LGE‐MRI at 1 day, *n* = 14 and 14 days, *n* = 10) two‐tailed Student's *t*‐tests and one‐way ANOVA with Tukey's post hoc test (1 h vs 1 day vs 14 days). Correlations 1 h versus 1 day in Figure 4c and MEMRI versus LGE‐MRI at 1 and 14 days in Figure 4d were calculated using Pearson correlation.

## Conflict of Interest

The authors declare no conflict of interest.

## Supporting information

Supporting Information

## Data Availability

The data that support the findings of this study are available from the corresponding author upon reasonable request.
