# Supplementary material for: Myocardial Viability Imaging using Manganese‐Enhanced MRI in the First Hours after Myocardial Infarction
Source: Adv Sci (Weinh). 2021 Apr 2;8(11):2003987. doi: 10.1002/advs.202003987 (PMC8188227; doi:10.1002/advs.202003987)
Supplement: Supplementary file 1 — Supporting Information [file ADVS-8-2003987-s001.pdf]

© 2021 Wiley-VCH GmbH

ADVANCED SCIENCE
Open Access

Supporting Information

for *Adv. Sci.,* DOI: 10.1002/advs.202003987

Myocardial viability imaging using manganese-enhanced MRI in the first hours after myocardial infarction

*Nur Hayati Jasmin, May Zaw Thin, Robert D Johnson, Laurence H Jackson, Thomas A Roberts, Anna L David, Mark F Lythgoe, Philip C Yang, Sean M Davidson, Patrizia Camelliti and Daniel J Stuckey**

Supporting Information

Myocardial viability imaging using manganese-enhanced MRI in the first hours after myocardial infarction

*Nur Hayati Jasmin, May Zaw Thin, Robert D Johnson, Laurence H Jackson, Thomas A Roberts, Anna L David, Mark F Lythgoe, Philip C Yang, Sean M Davidson, Patrizia Camelliti and Daniel J Stuckey**

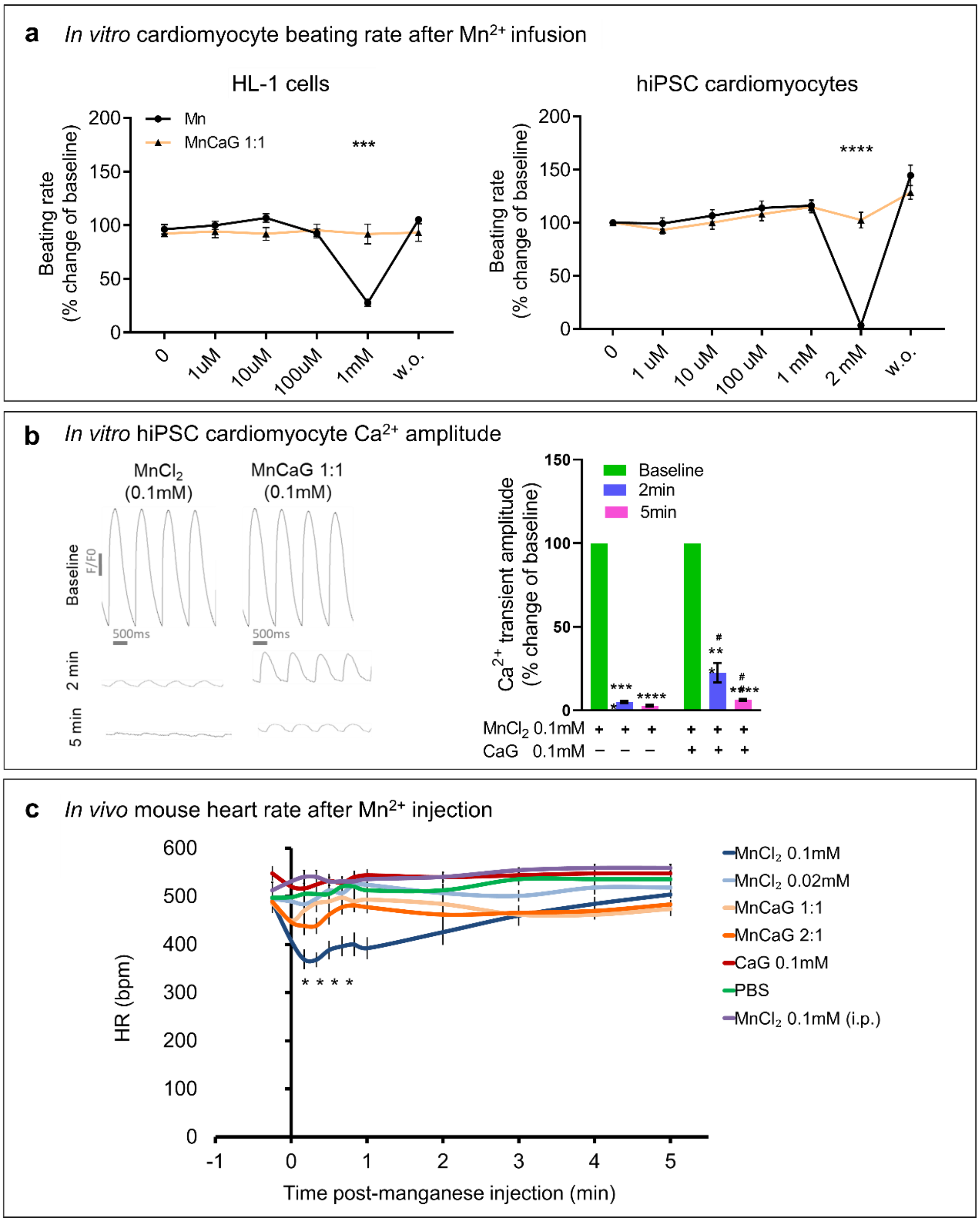

Figure. S1 Effects of manganese on *in vitro* cardiomyocyte beating rate and Ca^{2+} transients, and *in vivo* mouse heart rate. (a) Relationship between percentage change in beating rate of HL-1 and hiPSC cardiomyocytes with increasing concentration of $MnCl_2$ or MnCaG 1:1. Data were analysed by unpaired two tailed t-test (n=4, ****p*=0.0006; n=9, *****p*<0.0001). **(b)** Representative optical Ca^{2+} transient traces from hiPSC cardiomyocytes at baseline and at 2 and 5 min after superfusion with 0.1mM $MnCl_2$ with/out CaG supplement. Ca^{2+} transient amplitude was reduced by 0.1mM $MnCl_2$ and partially restored by supplement with 0.1mM CaG (n=4). Data were analysed by paired two-tailed Student's t-test to compare with baseline ****p*=0.0009 *****p*<0.0001 and unpaired two-tailed Student's t-test to compare $MnCl_2$ to $MnCl_2$+CaG^- #*p*=0.0247 ###*p*=0.0042). **(c)** Time course of heart rate showed a significant reduction after *i.v.* infusion of 0.1mM $MnCl_2$ (n=6) at 10 (*p*=0.044), 20 (*p*=0.019), 30 (*p*=0.016), and 40 seconds (*p*=0.041) which can be reversed when Mn^{2+} is supplemented with CaG (n=6). Mean value ± standard error mean (SEM). Data were analysed by two-way repeated measures ANOVA followed by Dunnett's post hoc test (compared to baseline). Raw data is presented in Table S2.

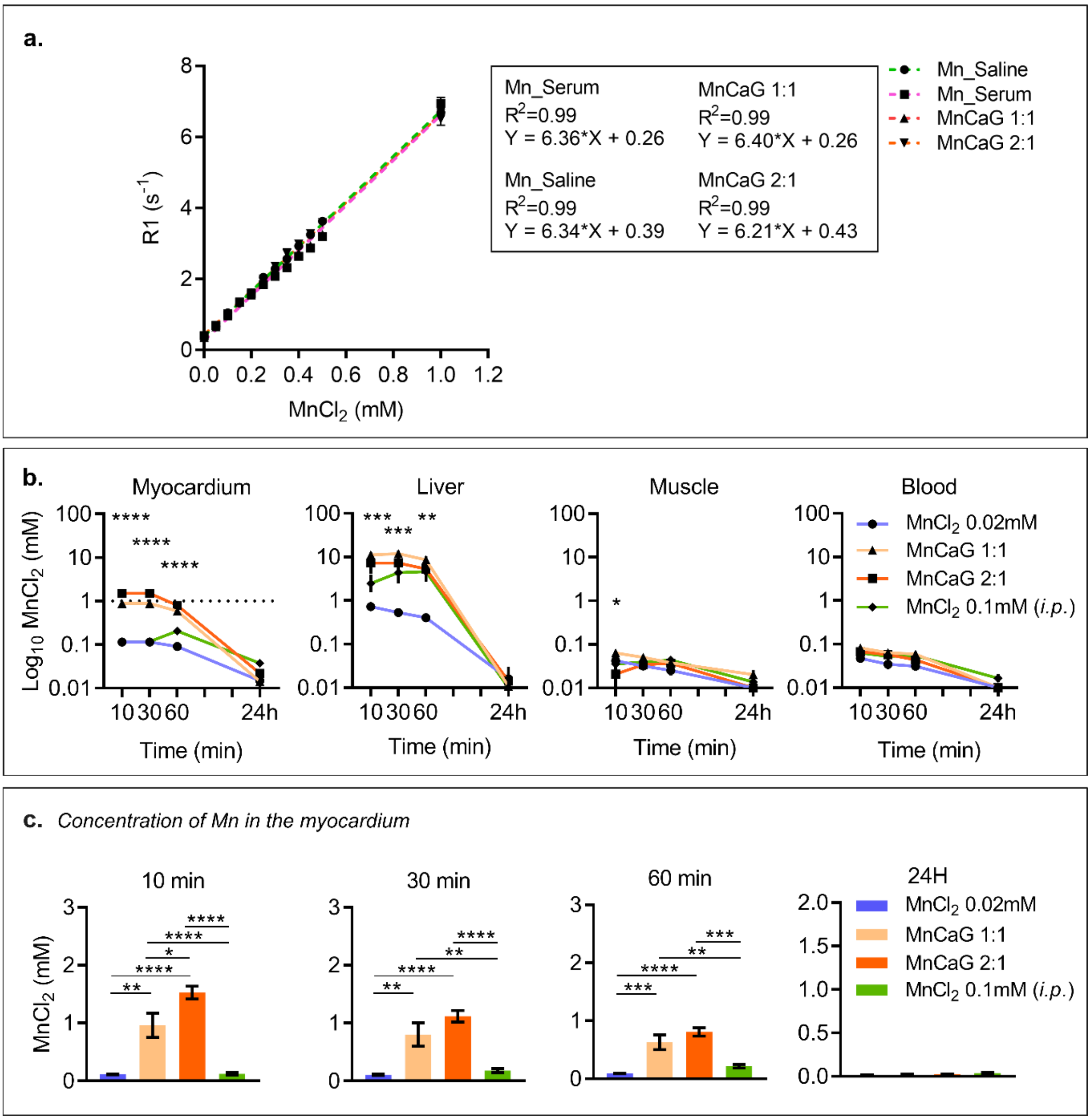

Figure. S2 Time-course of manganese biodistribution. (a) Phantom study of longitudinal relaxation rate constant (R1) versus Mn^{2+} concentration showed a strong linear correlation. The apparent value (round) and intrinsic value (dotted line) are shown. Data were analysed using linear regression analysis. **(b)** *In vivo* time course of estimated manganese concentrations in the liver, myocardium, blood and muscle at 10, 30, 60 minutes and 24-hour post-Mn-based contrast agent injection ($MnCl_2$ 0.02mM, MnCaG1:1, MnCaG2:1, and $MnCl_2$ 0.1mM *i.p.*). (n=5). Data were analysed by one-way ANOVA. Myocardium 10min, 30min, 60min (****p<0.0001) and 24h (*p=0.0162); Liver 10min (***p=0.0002), 30min (***p=0.0006), and 60min (*p=0.0064); Muscle 10min (*p=0.0193). **(c)** *In vivo* estimates of the concentration of Mn^{2+} in the myocardium at 10, 30, 60 minutes and 24-hour post injections. Data were analysed by one-way ANOVA followed by Tukey's post hoc test (*p<0.05, **p<0.01, ***p<0.001, and ****p<0.0001).

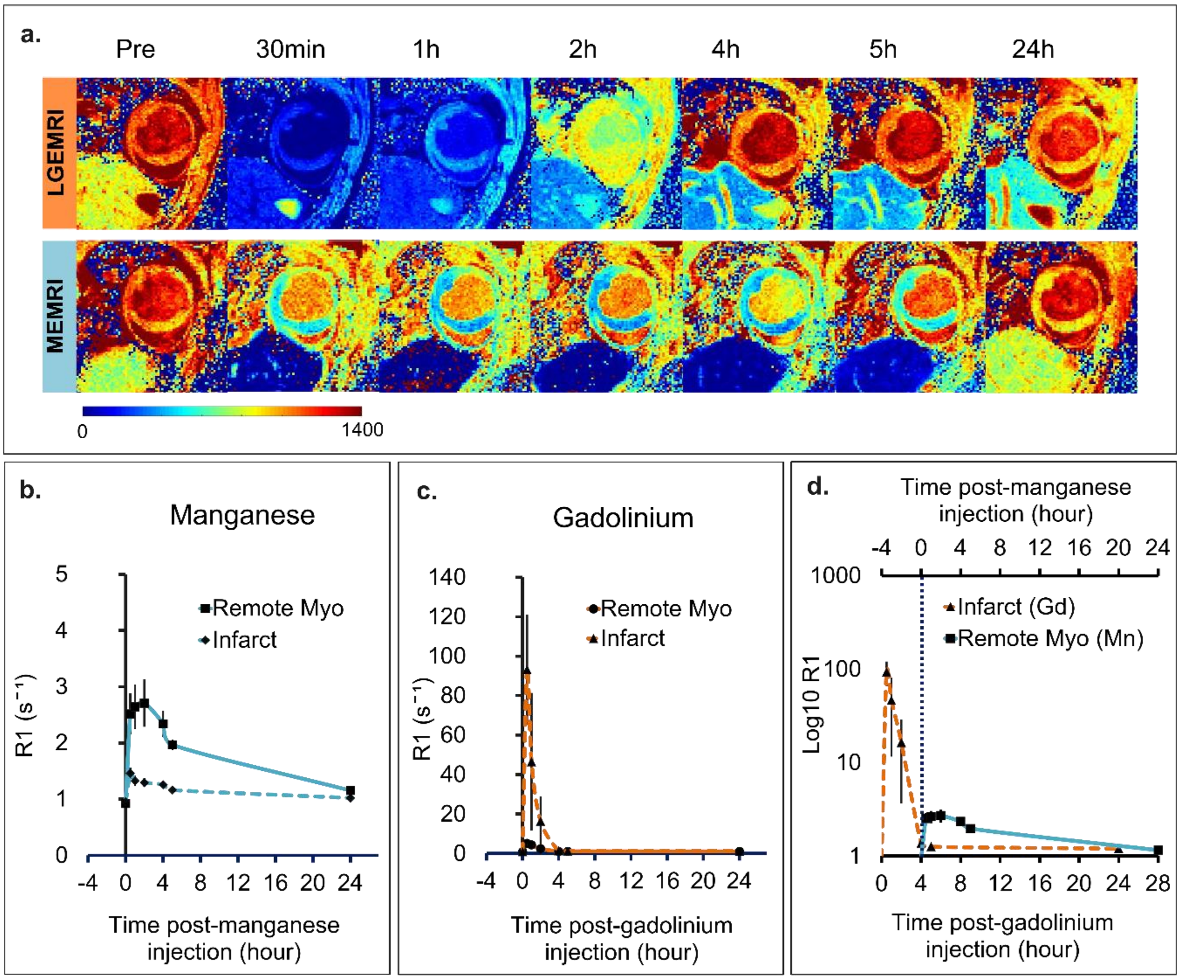

Figure. S3 Gadolinium and manganese washout rates from infarcted and remote areas of myocardium. (a) Representative T1 maps acquired over the time course of Gd-DTPA and $MnCl_2$ administration and washout. **(b)** R1 value (mean ± SEM) reflecting the amount of gadolinium in the remote myocardium and infarct region. The peak accumulation of gadolinium occurred at 30 minutes post-Gd injection in all three regions. **(c)** R1 value reflecting the amount of manganese in the remote myocardium and infarct region. The peak manganese uptake occurred at 2-hours post-Mn injection in the remote myocardium and 30 minutes post-Mn in the infarct region. **(d)** Data suggest that late gadolinium enhancement imaging should be performed prior to MEMRI, with a wait of at least 6 half-lives (4 hours) before injecting manganese to ensure the independence of contrast-enhancement patters. Data also indicate that, LGE is best performed within 30 minutes post-Gd injection and MEMRI performed at 60 minutes post-$MnCl_2$ injection.

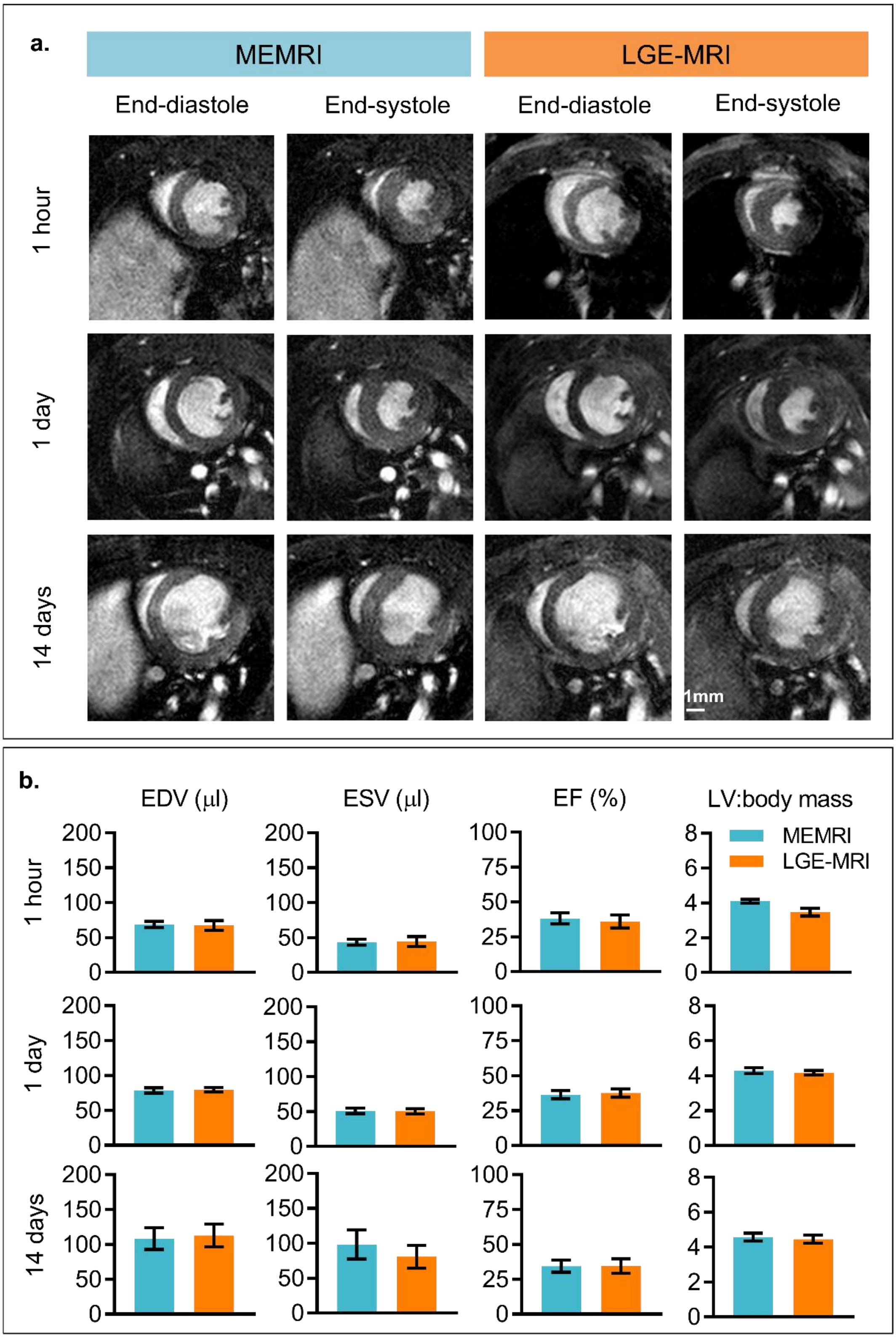

Figure. S4 Cardiac function of infarcted mice that underwent MEMRI and LGE-MRI. (a) End-diastolic and end-systolic cine-MRI images from both MEMRI and LGE-MRI groups at 1 hour (n=7), 1 day (n=14), and 14 days (n=10) post-MI. Scale bar, 1mm. **(b)** Corresponding Cine-MRI measurements of cardiac morphology and contractility. EDV indicates end-diastolic volume; EF, ejection fraction; ESV, end-systolic volume; and LV, left ventricle. No significant differences were found at any time point post-MI between groups. Data were analysed using unpaired (MEMRI vs. LGE-MRI at 1 hour) and paired (MEMRI vs. LGE-MRI at 1 day and 14 days) two-tailed t-tests and one-way ANOVA followed by Tukey's post hoc test (LGE-MRI, 1 hour vs. 1 day and 1 hour vs. 14 days).

Table. S1 Cardiac contractility following administration of different formulations of manganese contrast agents.

Time (s)	$MnCl_2$ 0.1mM (n=6)	$MnCl_2$ 0.02mM (n=4)	MnCaG 1:1 (n=6)	MnCaG 2:1 (n=6)	CaG (n=6)	PBS (n=3)	$MnCl_2$ 0.1mM (*i.p.*) (n=5)
Pre	38.21 ± 2.5	39.75 ± 6.1	38.55 ± 2.3	38.70 ± 2.3	40.75 ± 2.7	40.69 ± 1.1	39.08 ± 1.7
0	2.21 ± 0.7 P=0.0003	33.13 ± 3.7	49.37 ± 4.8	30.17 ± 5.8	56.65 ± 3.7 P=0.0003	39.19 ± 0.6	39.27 ± 0.8
10	1.57 ± 0.2 P=0.0002	12.19 ± 5.8 P=0.0235	51.35 ± 3.8	23.59 ± 7.5	49.43 ± 4.1 P=0.0498	39.72 ± 2.3	41.35 ± 1.3
20	1.29 ± 0.5 P=0.0002	32.96 ± 6.0	51.62 ± 3.8	28.03 ± 6.3	46.71 ± 3.6 P=0.0415	40.86 ± 2.5	41.51 ± 0.5
30	6.23 ± 3.2 P=0.0055	44.22 ± 2.7	53.14 ± 3.2 P=0.0309	41.54 ± 7.4	46.48 ± 4.1	40.71 ± 2.1	41.49 ± 1.2
40	10.89 ± 7.5	44.13 ± 3.1	53.94 ± 2.8 P=0.0182	46.81 ± 3.5	45.18 ± 3.0 P=0.0027	40.33 ± 2.8	41.34 ± 0.8
50	12.12 ± 8.1	45.27 ± 3.0	53.37 ± 2.7 P=0.0092	50.46 ± 3.7 P=0.01	45.82 ± 2.9 P=0.0498	40.71 ± 1.4	42.47 ± 0.5
60	16.51 ± 8.1	43.62 ± 2.2	50.81 ± 1.1 P=0.0070	51.04 ± 3.6 P=0.0245	45.29 ± 2.5 P=0.0415	41.38 ± 2.5	42.41 ± 0.2
120	34.30 ± 9.9	44.15 ± 2.9	46.26 ± 2.7	50.35 ± 4.3 P=0.025	41.48 ± 3.4	40.51 ± 2.6	42.31 ± 0.9
180	42.08 ± 4.6	43.52 ± 3.1	46.33 ± 2.5	48.28 ± 2.8 P=0.0019	40.54 ± 3.3	41.94 ± 3.3	41.93 ± 0.9
240	45.54 ± 3.2	46.76 ± 3.0	46.57 ± 3.2	49.88 ± 3.2 P=0.0138	39.89 ± 3.4	43.13 ± 3.4	41.49 ± 1.2
300	46.56 ± 1.4	46.81 ± 3.6	45.80 ± 1.6 P=0.0129	49.50 ± 2.7 P=0.0024	41.38 ± 3.4	44.14 ± 3.2	41.23 ± 1.5

Values represent mean ± SEM. $MnCl_2$, Manganese(II) chloride; MnCaG 1:1, manganese to calcium-gluconate ratio (1:1); MnCaG 2:1, manganese to calcium-gluconate ratio (2:1); CaG, calcium-gluconate; PBS, Phosphate-buffered saline; *i.p.*, intraperitoneal injection. Data were analysed using two-way repeated measures ANOVA followed by Dunnett's post hoc test (compared to baseline). Boxes marked red and green indicate cardiac contractility below and above the normal level, respectively.

Table S2. Heart rate following administration of different formulations of manganese contrast agents.

Time (sec)	$MnCl_2$ 0.1mM (n=6)	$MnCl_2$ 0.02mM (n=4)	MnCaG 1:1 (n=6)	MnCaG 2:1 (n=6)	CaG (n=6)	PBS (n=3)	$MnCl_2$ 0.1mM (*i.p.*) (n=5)
Pre	489 ± 23	495 ± 11	485 ± 19	489 ± 24	548 ± 16	497 ± 8	513 ± 16
0	407 ± 14	489 ± 10	450 ± 18	447 ± 20	521 ± 13	498 ± 16	531 ± 17
10	369 ± 21 P=0.0440	483 ± 11	470 ± 17	439 ± 19	517 ± 13	505 ± 16	541 ± 14
20	368 ± 16 P=0.0191	495 ± 11	485 ± 18	439 ± 17	524 ± 13	505 ± 16	541 ± 14
30	388 ± 20 P=0.0163	512 ± 10	489 ± 17	462 ± 20	532 ± 15	505 ± 16	531 ± 9
40	396 ± 21 P=0.0413	507 ± 6	497 ± 20	478 ± 16	528 ± 14	520 ± 8	531 ± 9
50	400 ± 24	524 ± 12	489 ± 19	482 ± 14	549 ± 14	520 ± 21	531 ± 9
60	392 ± 23	524 ± 12	493 ± 14	478 ± 13	544 ± 13	513 ± 13	536 ± 7
120	426 ± 27	507 ± 6	484 ± 15	462 ± 9	549 ± 13	513 ± 13	541 ± 9
180	460 ± 22	501 ± 12	462 ± 12	466 ± 10	544 ± 13	536 ± 13	555 ± 5
240	485 ± 15	518 ± 15	462 ± 10	470 ± 13	548 ± 15	536 ± 13	559 ± 7
300	504 ± 8	518 ± 15	474 ± 14	483 ± 8	548 ± 15	536 ± 13	559 ± 7

Values represent mean ± SEM. Mn indicates manganese; MnCaG 1:1, manganese ratio calcium-gluconate 1:1; MnCaG 2:1, manganese ratio calcium-gluconate 2:1; CaG, calcium-gluconate; PBS, Phosphate-buffered saline; *i.p.*, intraperitoneal injection. Data were analysed using paired two-way ANOVA with Dunnett's correction (compared to baseline). Boxes marked red indicate heart rates below the normal level.
